# Evaluating metal cookware as a source of lead exposure

**DOI:** 10.1038/s41370-024-00686-7

**Published:** 2024-05-21

**Authors:** Katie M. Fellows, Shar Samy, Stephen G. Whittaker

**Affiliations:** 1 Hazardous Waste Management Program in King County, Seattle, WA USA; 2 Department of Environmental and Occupational Health Sciences, University of Washington, Seattle, WA USA

**Keywords:** Lead, Global marketplace, Metal exposure, Cookware

## Abstract

**Background:**

We previously demonstrated that aluminum cookware brought from Afghanistan by resettled families as well as some aluminum cookware available for purchase in the United States represent a previously unrecognized source of lead exposure. However, the extent to which this cookware represents a source of lead exposure to other United States residents is unclear.

**Objectives:**

To test additional cookware for lead content and its propensity to leach lead and other toxic metals. This will further our understanding of the extent to which this cookware represents a lead poisoning risk in the United States and elsewhere.

**Methods:**

We screened an additional 28 pieces of aluminum cookware and 5 brass items for lead content using an X-ray fluorescence (XRF) analyzer and used our leachate method to estimate the amount of lead that migrates into food. We also tested 17 additional stainless steel items to determine whether they would be safer alternatives.

**Results:**

Many aluminum cookware products contained in excess of 100 parts per million (ppm) of lead. Many also leached enough lead under simulated cooking and storage conditions to exceed recommended dietary limits. One hindalium appam pan (an Indian frying pan/wok) leached sufficient lead to exceed the childhood limit by 1400-fold. Brass cookpots from India also yielded high lead levels, with one exceeding the childhood limit by over 1200-fold. In contrast, stainless steel cookware leached much lower levels of lead.

**Impact:**

Aluminum and brass cookware available for purchase in the United States represents a previously unrecognized source of lead exposure.

## Introduction

### Previous lead in cookware study

Our 2022 study demonstrated that some aluminum cookware brought from Afghanistan by resettled families, as well as some aluminum cookware available for purchase in the United States, contained very high lead levels and that the lead readily migrates into a weak acidic solution that mimics low pH foodstuffs [1]. The previous study was prompted by the high prevalence of blood lead levels (BLLs) above the Center for Disease Control and Prevention’s (CDC’s) blood lead reference value (BLRV) in Afghan refugee children resettled in King County, WA [2]. This finding led to in-home environmental assessments at the primary residences of Afghan children with BLLs ≥ 5 micrograms per deciliter of whole blood (μg/dL). The intervention included X-ray fluorescence (XRF) screening for lead content and revealed that aluminum cookware brought by families from Afghanistan (including traditional Afghan pressure cookers and cook pots and pans) frequently contained lead levels in the hundreds of parts per million (ppm) and occasionally in the thousands of ppm.

Acting upon these in-home findings, our previous study reported comprehensive XRF screening on 40 pieces of aluminum cookware for lead content in the laboratory and the use of a novel leachate method to estimate the amount of lead that migrates into food. We also tested five pieces of stainless steel cookware to determine whether they would be safer alternatives [1]. We determined that all the traditional Afghan pressure cookers and many aluminum cook pots and pans contained high lead levels. The highest detected lead concentrations were found in the pressure relief vent pipes in the traditional pressure cookers (up to 69,000 ppm). The leachate tests revealed that 27 of the 40 pieces of aluminum cookware yielded sufficient lead to exceed the Food and Drug Administration’s (FDA’s) Interim Reference Level (IRL) for children, which is the recommended maximum daily dietary intake of lead from food [3]. Of particular concern was that enough lead leached from all 15 aluminum cookware items donated by local Afghan families to exceed FDA’s childhood IRL. In contrast, none of the stainless steel cookware leached lead in excess of the childhood IRL, suggesting that stainless steel is a safer alternative.

### Cookware definition

Note that we use the term “cookware” to describe food preparation equipment used on a stove or range cooktop, including pressure cookers, fry pans, sauté pans, sauce pans, woks, braziers, stock pots, fryer pots, steamers, calderos, and double boilers. Cookware imported from India and evaluated in this present study included examples of a “kadai,” (similar to a wok), “uruli” (cooking bowl), “tadka pan” (spice heating pan), “appam pan” (frying pan/wok), “idli maker” (steamer pot), “tope” (sauce pan), and “handi” (bean pot).

### Lead in cookware as a health hazard

Childhood lead poisoning is one of the most common pediatric health problems worldwide, although lead exposures and resulting disease are almost entirely preventable. Children are at particular risk for serious health effects from this toxic metal at any level of exposure [4]. Numerous studies have documented that low-level lead exposures can affect a child’s neurological development, with severe impacts on learning, intelligence, and behavior [5]. Exposures to lead and cadmium are also associated with cardiovascular disease [6].

The public health and economic costs associated with childhood lead poisoning are staggering. In the United States alone, the net benefit of implementing effective lead hazard controls is estimated to range between $181 and $296 billion, where the estimated costs for intervention range from $1.2 to $11 billion. Further, the estimated lost earning potential associated with childhood lead poisoning in low- and middle-income countries (LMICs) is estimated to be $977 billion annually [7].

Several studies have demonstrated that aluminum cookpots are important sources of lead exposure in some LMICs [8–17]. Manufacturing may be conducted by artisans, who typically produce uncoated, non-anodized cookware sometimes made from discarded scrap metal [8, 12, 15, 16]. This practice has resulted in lead poisoning in surrounding communities [18, 19]. However, lead contamination of aluminum cookware may also occur in more industrial-scale manufacturing if the facility uses primarily scrap metal or does not perform adequate quality control on its source material.

### Present study

Our previous study suggested that some aluminum cookware represents an important source of lead exposure to our local Afghan refugee community. We also proposed that stainless steel is a safer alternative. However, we had not tested enough products to determine the extent to which lead-containing cookware represents a lead exposure source for the United States population as a whole. We also did not generate sufficient data to be further assured that stainless steel is a safer alternative.

When formulating this present investigation, we discovered a study conducted in India that identified cookware as a source of lead exposure [20]. This study described the use of hindalium, an aluminum alloy manufactured in India. Consequently, this present study included aluminum cookware imported from India. We also noted the availability of Indian cookware manufactured from brass (a lead-containing alloy) and included several brass cookware items in our current study.

Our goals of this present study were to learn more about cookware used by diverse newcomer populations and long-term United States residents by (1) measuring the lead content of additional cookware available for purchase from online retailers, (2) measuring the amount of lead that leaches from additional cookware under simulated cooking and storage conditions, and (3) further evaluating whether stainless steel cookware represents a safer alternative.

## Methods

### Acquisition of cookware

We acquired an additional 51 pieces of cookware for analysis between September 2021 and July 2023. This cookware was purchased from several online marketplaces, including Amazon and Etsy. To identify potentially safer alternatives, we also purchased stainless steel cookware found within the same marketplaces. Information on the cookware such as description/type of cookware, primary material, and country of origin was determined through the item’s product listing, packaging materials, and identifying information on the cookware itself; no efforts were made to verify the accuracy of the information provided by the seller and/or manufacturer.

### XRF analysis

We conducted lead screening between October 2021 and July 2023 using a handheld Bruker S1 Titan XRF analyzer (Bruker Corporation, Billerica, MA). Methodological details are provided in our previous paper, including determining the XRF analyzer’s response to a series of lead standards in an aluminum alloy matrix [1]. In this current paper, we present the XRF analyzer’s response to lead standards in stainless steel, to ensure that the instrument is appropriately calibrated for this matrix (see Table S-1 and Fig. S-1).

### Leachate analysis

All leachates were quantitatively processed for lead concentration by inductively coupled plasma–mass spectrometry (ICP–MS) as described previously [1]. Briefly, the ICP–MS (Agilent 7900, Santa Clara CA) was operated with collision cell gas (He mode) and a matrix-matched calibration was performed to quantify leachates.

As described in our previous work [1], we used the FDA’s IRLs for lead as the benchmarks to evaluate the estimated lead doses from consuming 250 mL of liquid after bringing 4% acetic acid to a simmer for 15 min and then after allowing the liquid to sit in the cookware for 24 h. In 2022, the FDA lowered its IRL for children from 3 to 2.2 micrograms per day (µg/day). Similarly, the IRL for people of childbearing age was lowered from 12.5 to 8.8 µg/day [21]. These changes reflect the CDC’s lowering of the BLRV for children from 5 to 3.5 µg/dL in 2021 [22].

As detailed in our previous work [1], prior to experimentation, cookpots were washed with tap water and a mild dish soap, triple rinsed with ultra-pure deionized (DI) water (18.2 MΩ) and allowed to dry. Vessels were filled with diluted acetic acid (Glacial, Fisher TraceMetal^TM^ Grade) in DI water (4% v/v), then heated to a simmer using a cast iron electric burner (Cuisinart Model CB-60P1) at the highest temperature setting. Details about aliquot sampling, storage, preservation, and analysis can be found in the previous paper.

## Results

### Aluminum and brass cookware

**Fig. 1:**
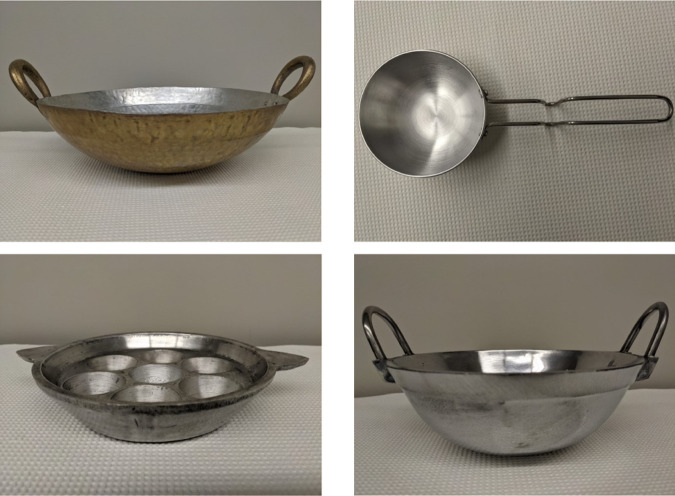
Examples of Indian cookware purchased for testing. Top left: Brass kadai (Pot #51). Top right: Hindalium/indolium tadka pan (Pot #53). Bottom left: Hindalium/indolium appam pan (Pot #54). Bottom right: Hindalium/indolium kadai (Pot #55).

We conducted XRF analyses and leachate testing on 28 pieces of aluminum cookware, 8 of which were manufactured in India. We also tested five brass items, four of which originated from India and one from Thailand (see Table 1). Examples of this cookware are shown in Fig. 1.

**Table 1:** XRF and leachate results for aluminum and brass cookware.

Pot #	Description	Primary material	Country of origin	Median Pb level (XRF, ppm)	Range of Pb levels (XRF, ppm)	Lead leachate conc. at 15 min (μg/mL)	Lead dose/serving at 15 min (μg/250 mL)	Lead leachate conc. at 24 h (μg/mL)	Lead dose/serving at 24 h (μg/250 mL)
50	Idli maker	Hindalium/indolium	India	626	(81, 1463)	0.0182	4.55^b^	0.132	33^c^
51	Kadai	Brass	India	5049	(128, 47,191)	0.766	192^c^	1.34	335^c^
52	Uruli/kadai	Hindalium/indolium	India	256	(103, 290)	0.00434	1.09	0.0268	6.7^b^
53	Tadka pan	Hindalium/indolium	India	290	(0, 306)	0.00389	0.973	0.135	33.8^c^
54	Appam pan	Hindalium/indolium	India	716	(603, 794)	0.0144	3.6^b^	12.3	3080^c^
55	Kadai	Hindalium/indolium	India	756	(15, 813)	0.0245	6.13^b^	0.926	232^c^
56	Unknown	Hindalium/indolium	India	517	(362, 636)	0.0157	3.93^b^	1.05	263^c^
57	Caldero	Aluminum alloy	Colombia	13	(0, 71)	0.00092	0.23	0.0185	4.63^b^
58	Idli maker	Food-grade aluminum	India	715	(308, 1327)	0.028	7^b^	0.27	67.5^c^
59	Caldero	Aluminum alloy	Colombia	8^a^	(0, 15)	0.00075	0.188	0.0157	3.93^b^
60	Caldero	Aluminum alloy	Colombia	9^a^	(0, 15)	0.0006	0.15	0.0167	4.18^b^
61	Caldero	Aluminum alloy	Colombia	7^a^	(0, 17)	0.0006	0.15	0.0149	3.73^b^
62	Kadai	Hindalium/indolium	India	528	(0, 624)	0.0457	11.4^c^	1.36	340^c^
80	Stock pot	Aluminum alloy	Unknown	9	(0, 38)	0.00005	0.0125	0.00023	0.0575
81	Cookpot	Aluminum alloy	Thailand	14	(0, 27)	0.00183	0.458	0.0154	3.85^b^
82	Stock pot	Aluminum alloy	USA	11^a^	(0, 19)	0.0001	0.025	0.00187	0.468
83	Stock pot	Aluminum alloy	USA	51	(0, 83)	0.00014	0.035	0.0007	0.175
84	Dutch oven	Aluminum alloy	USA	0^a^	(0, 17)	0.00083	0.208	0.00255	0.638
85	Stock pot	Aluminum alloy	El Salvador	15	(0, 27)	0.00118	0.295	0.0105	2.63^b^
86	Pressure cooker	Aluminum alloy	China	8^a^	(0, 147)	0.00053	0.133	0.004	0.898
87	Stock pot	Aluminum alloy	USA	11^a^	(0, 15)	0.00014	0.035	0.003	0.793
88	Stock pot	Aluminum alloy	China	50	(0, 94)	0.00105	0.263	0.010	2.55^b^
89	Cookpot	Aluminum alloy	China	9^3^	(0, 26)	0.00045	0.113	0.001	0.243
90	Cookpot	Aluminum alloy	Vietnam	0^a^	(0, 34)	0.00012	0.03	0.0003	0.0825
91	Cookpot	Aluminum alloy	China	0^a^	(0, 20)	0.00013	0.0325	0.00024	0.06
92	Cookpot	Aluminum alloy	China	13	(0, 26)	0.013	3.25^b^	0.0207	5.18^b^
93	Pressure cooker	Aluminum alloy	Afghanistan	538	(0, 48,193)	0.183	45.8^c^	3.96	990^c^
94	Pressure cooker	Aluminum alloy	Afghanistan	605	(0, 64,852)	0.194	48.5^c^	4.63	1158^c^
95	Pressure cooker	Aluminum alloy	Afghanistan	548	(0, 51,021)	0.131	32.8^c^	3.12	780^c^
96	Wok	Brass	Thailand	0^a^	(0, 0)	0.00455	1.13	0.00549	1.38
97	Sauce pan	Brass	India	0^a^	(0, 1182)	0.079	19.8^c^	0.129	32.3^c^
98	Tope	Brass	India	1343	(1012, 1544)	0.418	105^c^	0.728	182^c^
100	Pital pot	Brass	India	3611	(2859, 4585)	5.72	1430^c^	10.8	2700^c^

^a^More than 50% of XRF measurements <LOD.

^b^Estimated dose ≥ child IRL (2.2  µg/day) and <adult IRL (8.8  µg/day).

^c^Estimated dose ≥ adult IRL (8.8  µg/day).

The XRF screening and leachate results for aluminum and brass cookware are presented in Table 1. The highest lead concentrations were found in the brass kadai (Pot #51), where the median concentration was 5049 ppm (range: 128–47,191 ppm). Lead concentrations in excess of 10,000 ppm were detected in the kadai’s handles. The second and third highest median lead concentrations were found in a brass pital pot (Pot #100: 3611 ppm) and a brass tope (Pot #98: 1343 ppm), respectively. All three of these brass items originated from India.

Among the aluminum cookware, the highest lead concentrations were detected in Afghan pressure cookers and hindalium products. Pot #58, an idli maker made from “food-grade aluminum,” had lead levels similar to the hindalium items.

When sampled 15 min after the acetic acid had been brought to a boil (i.e., *t* = 15 min), lead concentrations in leachate ranged from 0.00005 micrograms per milliliter (µg/mL) (Pot #80) to 5.72 µg/mL (Pot #100). Estimated daily lead doses from one serving in these cookpots ranged from 0.0125 to 1430 µg/day. The doses from 14 items of cookware exceeded the childhood IRL and 8 also exceeded the adult IRL (i.e., for people of childbearing age). Note that the brass pital pot (Pot #100) yielded the highest estimated daily lead dose after 15 min.

Additional leaching occurred in all cookware after 24 h at room temperature (i.e., *t* = 24 h), by as much as 850-fold (Pot #54). Lead concentrations in leachate ranged from 0.00023 µg/mL (Pot #80) to 12.3 µg/mL (Pot #54). Estimated daily lead doses in this cookware ranged from 0.0575 to 3075 µg/day. The doses from 23 items of cookware exceeded the childhood IRL and 14 also exceeded the adult IRL. Among the aluminum items, the Indian cookware and Afghan pressure cookers leached the highest concentrations of lead. The hindalium appam pan (Pot #54) and the brass pital pot (Pot #100) yielded the highest daily doses after 24 h, with exceedances of the childhood IRL of 1400-fold and 1200-fold, respectively.

### Stainless steel cookware

We also conducted XRF and leachate analyses on 17 stainless steel items, including 6 pressure cookers, 3 stock pots, 6 sauce/sauté pans, and 2 skillets. One pressure cooker was an electric model that is favored by the local Afghan community as an alternative to traditional Afghan pressure cookers (Pot #99) (see Table 2).

**Table 2:** XRF and leachate results for stainless steel cookware.

Pot #	Description	Country of origin	Median Pb level (XRF, ppm)	Range of Pb levels (XRF, ppm)	Lead leachate conc. at 15 min (μg/mL)	Lead dose/serving at 15 min (μg/250 mL)	Lead leachate conc. at 24 h (μg/mL)	Lead dose/serving at 24 h (μg/250 mL)
63	Pressure cooker	India	1^a^	(0, 55,009)^b^	0.0003	0.075	0.00056	0.14
64	Pressure cooker	India	27^a^	(0, 53,294)^b^	0.00143	0.358	0.00159	0.398
65	Pressure cooker	Unknown	20^a^	(0, 70)	0.00015	0.0375	0.00018	0.045
66	Stock pot	China	5^a^	(0, 79)	0.00013	0.0325	0.00015	0.0375
67	Sauce pan	China	11^a^	(0, 53)	0.00022	0.055	0.00026	0.065
68	Frying pan	China	17^a^	(0, 46)	0.0006	0.15	0.00064	0.16
69	Sauté pan	China	11^a^	(0, 62)	0.00024	0.06	0.00024	0.065
70	Stock pot	China	36^a^	(0, 75)	0.00011	0.0275	0.00012	0.03
71	Sauce pan	China	17^a^	(0, 97)	0.00024	0.060	0.00026	0.065
72	Sauce pan	China	22^a^	(0, 69)	0.00033	0.0825	0.00036	0.09
73	Stock pot	Unknown	0^a^	(0, 67)	0.0003	0.075	0.0003	0.075
74	Sauce pan	Unknown	0^a^	(0, 41)	0.00023	0.0575	0.00026	0.065
75	Sauce pan	Unknown	0^a^	(0, 58)	0.00069	0.173	0.00075	0.188
76	Skillet	Unknown	0^a^	(0, 28)	0.00088	0.22	0.0009	0.225
77	Pressure cooker	India	8^a^	(0, 4124)^b^	0.00164	0.41	0.00208	0.52
78	Pressure cooker	India	17^a^	(0, 4040)^b^	0.00031	0.0775	0.00163	0.408
99	Pressure cooker (electric)	China	15^a^	(0, 87)^b^	0.00021	0.0525	0.00025	0.0625

^a^More than 50% of XRF measurements <LOD.

^b^Highest lead concentration detected in the pressure cooker vent pipe.

The median lead concentrations ranged from below the LOD to 36 ppm (Pot #70). The two highest lead concentrations (53,294 and 55,009 ppm in Pots #64 and #63, respectively) were found in the pressure cooker vent pipes in models imported from India.

At *t* = 15 min, lead leachate levels ranged from 0.00011 to 0.00164 µg/mL, corresponding to estimated daily doses of 0.0275 and 0.41 µg/day, respectively. At *t* = 24 h, lead leachate levels ranged from 0.00012 to 0.00208 µg/mL, corresponding to estimated daily doses of 0.03 to 0.52 µg/day. No stainless steel cookware leached sufficient lead to exceed childhood or adult IRLs.

## Discussion

The results of this study suggest that aluminum and brass cookware readily available for purchase in the United States contains high lead levels and leaches sufficient lead to represent a potentially important source of lead exposure. This lead-containing cookware appears to be manufactured overseas, where it is imported to the US and distributed for sale both online and in traditional retail outlets.

### Afghan pressure cookers

The findings from this present study confirmed that traditional Afghan pressure cookers contain very high lead levels and leach sufficient lead to exceed childhood IRLs. All nine Afghan pressure cookers we have tested (i.e., six from our previous study and three in this current work) yielded sufficient lead to exceed both the adult and childhood IRLs. Although several of the Afghan pressure cookers tested in the previous study were hand-carried from Afghanistan and donated for analysis, the pressure cookers in this present study were purchased from online retailers in the United States. Even though two of the pressure cookers analyzed in the present study are a different brand from others tested, they exhibited similar lead content and lead leachate levels. This finding suggests that lead contamination is independent of manufacturer and reflects the widespread use of lead-contaminated scrap aluminum in Afghanistan.

### Hindalium cookware from India

Every hindalium item contained high lead levels and the amount extracted during the leachate tests exceeded IRLs. We evaluated these items because aluminum cookware has been identified as a potential source of lead exposure in India [23]. In addition, India is one of the world’s largest producers of aluminum [24], and Indian cookware made from aluminum is readily available for purchase in the United States.

Hindalium is the trade name for an alloy comprised of aluminum, magnesium, manganese, chromium, silicon, etc., and is produced in India by the Hindustan Aluminium Corporation (also known as Hindalco) [25]. However, marketing materials for hindalium cookware often use similar terms, such as “indolium,” “indalium,” and “hindolium.” Despite attempts at correspondence with Indian authors of relevant papers and other articles, we were unable to verify from metallurgists or other technical specialists whether these terms refer to the same alloy. However, email correspondence with Indian cookware vendors suggested that the terms are used synonymously. (Note that “indolium” typically refers to an unrelated cationic substance, also known as 1H-Indol-1-iumyl) [26].

As stated previously, we became aware of hindalium via a 1998 study conducted in India, which evaluated the leachability of lead from pressure cookers [20]. This study concluded that while pressure cookers introduced lead into food, their contribution relative to the lead content of the foodstuffs being prepared was relatively low. However, this study used tap water and tamarind juice for extraction, which are likely less acidic than the 4% acetic acid used in our experiments (the pHs of the extractants were not provided in the paper).

Another study from India investigated migration of aluminum from indalium cookware and concluded that these vessels contributed significantly to the total daily intake of aluminum through foods [27]. The concentrations of lead and other toxic metals were not measured. Interestingly, concern about aluminum from cookware as a cause of Alzheimer’s disease and other neurological conditions is very common on the internet, including on Indian web sites. One example, published by a member of an ashram, specifically cautions against using hindalium cookware because of aluminum extraction into foods [28].

### Brass cookware from India

All the brass cookware from India contained very high lead levels and/or the amount extracted during the leachate tests exceeded IRLs.

We discovered brass cookware in our search for hindalium items to purchase. In India, brass is an increasingly common cooking material, and many consider it to confer health benefits [29, 30]. Brass is primarily an alloy of copper and zinc, and lead is frequently added to improve corrosion resistance and machinability [31]. Some brasses can contain up to 7% lead [32]. However, as is the case with aluminum, lead levels can potentially be higher without treatment of source material or adequate quality control in the recycling process.

### Stainless steel cookware

The stainless steel cookpot bodies contained very low lead levels and the amount extracted during the leachate tests did not exceed IRLs. However, all four stainless steel pressure cookers imported from India were fitted with a pressure relief vent pipe that contained high lead concentrations, with two exceeding 50,000 ppm. This finding is consistent with our previous study, where we speculated that vent pipes are comprised of brass or another lead-containing alloy. Nonetheless, no stainless steel cookpots yielded sufficient lead in leachate to exceed IRLs. The electric pressure cooker favored by the Afghan community as a safer alternative to lead-containing traditional pressure cookers (Pot #99) did not yield sufficient lead in the leachate test to exceed IRLs. However, a lead concentration of 87 ppm was detected in the vent pipe of this pressure cooker. Although vent pipes do not appear to contribute significant amounts of lead to the acetic acid solution, lead-containing components should not be used in cookware.

### Overview of lead extraction by cookware material

**Fig. 2:**
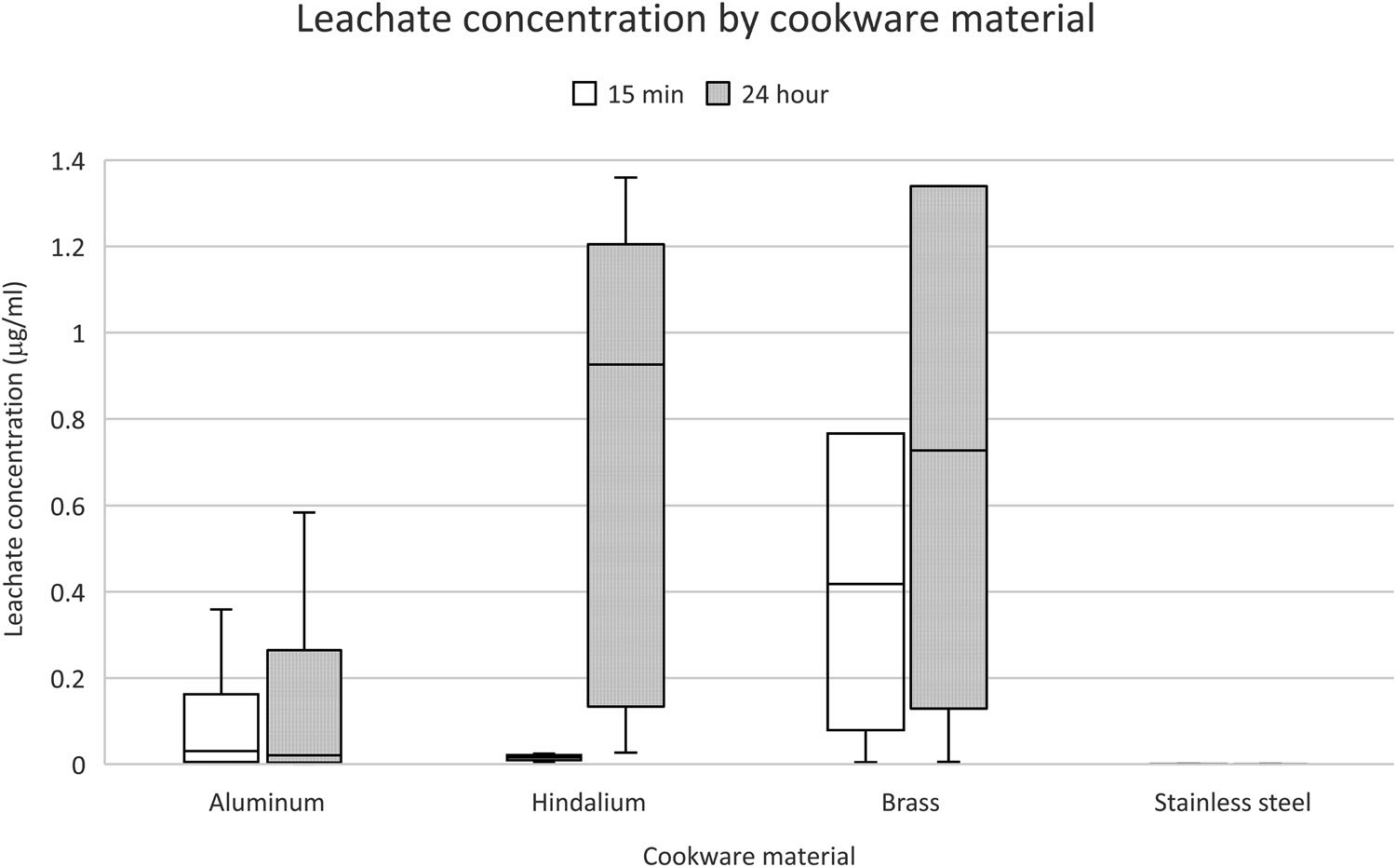
Leachate concentration by cookware material. Concentrations 15 min after simmering (15 min) are in white, whereas concentrations 24 h after sitting at room temperature (24 h) are in gray. The median concentration is depicted by the middle line; the lower and upper interquartile concentrations are depicted by the box, and the minimum and maximum concentrations are depicted by the lower and upper whiskers.

Figure 2 depicts the leachate results for all the cookware tested by our program, including cookware described in our previous study [1]. This complete dataset contains data for 96 individual pieces of cookware, manufactured primarily from aluminum (62 pieces), hindalium (7 pieces), brass (5 pieces), and stainless steel (22 pieces). This figure demonstrates that (1) no stainless steel cookware leached lead above levels of concern, even after 24 h; (2) for the other materials, significantly more lead was extracted after 24 h, compared to 15 min; and (3) leachate concentrations are typically significantly higher in hindalium and brass cookware than in other aluminum alloys.

### Strengths and limitations of the study

Our 2022 paper described the strengths and limitations of this type of study in detail, where strengths included the benefits of using an XRF analyzer to screen cookware. Evaluating our XRF analyzer’s response against a series of lead calibration standards in aluminum and stainless steel matrices ensured that the instrument was appropriately configured for this study. The importance of this practice was underscored when working with partners using different analyzer models, particularly when screening stainless steel items (their analyzers yielded false positive results). We found the following factors to be critical: (1) the measurement time must be of sufficient duration to reduce error and increase precision, (2) the appropriate calibration program must be used for the material being tested, (3) the analyzer responses against calibration standards should be critically evaluated, and (4) the spectra must be available for review – to evaluate the possibility of false positive results, especially near the instrument’s limit of detection.

Our development of a leachate test to estimate exposures that account both for cooking and storage of food in cookware was also a significant benefit of this study.

Limitations include the limited sample size of tested cookware, due to resource constraints. Consequently, while we can make generalizable statements about the hazards associated with preparing food in some aluminum, hindalium, and brass cookware, the extent to which other commonly available metallic cookware available in the United States marketplace represents a lead poisoning risk is unclear.

Our test method involved boiling the acetic acid for 15 min. However, we recently learned that the procedure employed by NSF International under its P390 standard (stovetop cookware for home use) involves simmering acetic acid for 30 min [33]. In addition, a recent recommendation from the FDA and a proposed certification from Clean Production Action specify simmering for 2 h [34, 35]. Consequently, our test method may extract less lead from cookware and therefore may be less health-protective than these other approaches. We also recognize that the degree to which lead is extracted from cookware is dependent on pH. However, it was beyond the scope of this present study to investigate the impact of using lower acetic acid concentrations, which may more accurately reflect the pH of many foods prepared in this cookware.

Our study did not examine the impact of cookware aging on extractable toxic metals. Surfaces that have become pitted or damaged with use could potentially release more lead. We also did not consider the potential contribution of lead from aluminum or brass utensils.

A significant uncertainty with our experimental design was the application of a leachate test to cookware that is typically used for frying or steaming foods at ambient pressures. Although we consider it inappropriate for any cookware to contain detectable levels of lead, a more representative approach for these items would be to measure the amount of lead extracted into heated edible oils or steam. However, relatively acidic sauces and other liquids may occasionally be added to the contents of frying vessels and then heated.

Finally, the assumption that both children and people of childbearing age consume 250 mL (i.e., 1 cup) per day from this type of cookware may under- or over-estimate their exposure.

## Conclusions and recommendations

To our knowledge, this study is the first to identify Indian cookware manufactured from aluminum and brass as a potentially important source of lead exposure in the United States. Our previous study identified the potential for significant lead exposures from traditional Afghan pressure cookers purchased in the US (confirmed in this present study), as well as from aluminum pots and pans hand-carried from Afghanistan (reportedly manufactured in India, Pakistan, and China [36]). Other lead-containing aluminum cookware was also available for purchase in the United States, with countries of origin including Colombia, Taiwan, and China. This latest study further confirms our previous hypothesis that stainless steel is a safer alternative to aluminum and brass for cookware. However, the use of lead-containing ancillary components, like vent pipes, should be prohibited.

Although our cookware studies have focused on lead content, it is important to note that many items contained relatively high levels of other toxic metals that were also extracted into the acetic acid solution. For example, XRF measurements for cadmium ranged from 0 to 5157 ppm and leachate levels ranged from 0.00001 to 0.093 µg/mL, corresponding to estimated daily doses of 0.0025 to 23.3 µg/day. Some of the highest Cd levels were found in Afghan pressure cookers and hindalium cookpots from India (unpublished observations). However, we are not aware of any guidelines or regulatory standards for cadmium in cookware.

The ready availability of cookware originating from multiple countries via the global marketplace and elsewhere indicates that (1) both newcomer populations and longer term residents of the United States (and other high-income countries) are exposed to lead via metal cookware, and (2) this cookware likely represents a significant contributor to lead poisoning in LMICs. Our previous publication described the relatively small-scale production of aluminum cookware by artisans in several LMICs. However, the ready availability of lead-containing cookware globally suggests that manufacturing using scrap aluminum also occurs in industrial operations at scale. A search of the online video platform, YouTube, confirmed this practice (https://www.youtube.com/results?search_query=aluminium+vessels+manufacturing). Of note is the use of engine parts and other aluminum scrap in the manufacture of the most popular brand of Afghan pressure cooker (https://www.youtube.com/watch?v=ManknFL2BIw&t=57s).

### National and local interventions

Upon learning about the potential for significant lead exposures from metal cookware, we notified the FDA in 2022, the United States agency that would have regulatory authority. The FDA subsequently issued an import alert for the most popular brand of Afghan pressure cooker, which specifies detention without physical examination [37]. Although this is a good first step, we recommend that the FDA issue additional import alerts for lead-containing metal cookware and actively intervene with distributors in the USA.

The FDA does not currently administer a lead standard for metal cookware, although it enforces a leachate lead limit for ceramicware and silver-plated hollowware [38, 39]. Therefore, we recommend that the FDA develop a national standard for lead in metal cookware, based on the leachate method for ceramicware.

In 2023, we informed the three principal online retailers in the United States – Amazon, Etsy, and eBay – that they were selling lead-containing cookware and requested that they no longer offer it for sale. As of July 2023, these companies removed most Afghan pressure cookers from their platforms. Given that promulgation of a new regulation by the FDA would likely be a lengthy process, we recommend that the FDA take the interim action of working with retailers and distributors to implement a voluntary lead content standard. Retailers and distributors could then require a lead-safe certification from their suppliers.

At the local level, we engaged with a community-based organization (the Afghan Health Initiative) to develop an outreach strategy for local Afghan refugees and immigrants. We co-developed a cookware exchange program, where families are provided electric pressure cookers in return for their traditional cookware. This program has proven popular: the electric pressure cooker model evaluated in this study has been adopted readily by this community. As of December 2023, we have provided almost 200 electric pressure cookers. We recommend that additional jurisdictions institute an exchange program to provide communities with safer cookware.

Recommendations provided in our previous paper remain salient. Namely, providing information about lead in cookware to newcomer communities in a culturally appropriate manner; sharing information with all United States residents about the presence of lead in some metal cookware; increasing the rate of blood lead testing among at-risk children; educating health care providers about the risks to children associated with BLLs at or near the BLRV, and providing sufficient resources to local health jurisdictions to effectively manage lead poisoning surveillance data and conduct interventions with at-risk children.

### Opportunities for global interventions

Approximately one in three children suffer from lead poisoning worldwide [40–42], with a disproportionate burden on children in LMICs. A 2023 evaluation of the global health burden of lead exposure in LMICs by the World Bank concluded that lead exposure is an environmental risk factor on par with PM2.5 ambient and household air pollution combined, and ahead of unsafe household drinking water, sanitation, and handwashing [43].

Food prepared in metal cookware may represent a previously unrecognized source of lead exposure, especially in LMICs [44, 45]. Additional resources should be provided by national governments and global aid organizations to identify lead-containing cookware and other consumer products available in LMICs.

The identification of lead-containing products imported to the United States marketplace indicates that these items may be contributing to lead exposures in their countries of manufacture, which are frequently LMICs. Therefore, as recommended by the New York City Department of Health and Mental Hygiene, a national repository of consumer product lead surveillance data should be developed [46]. This national dataset could be used to inform interventions both within the United States and globally.

In conclusion, considering that childhood lead poisoning is an almost entirely preventable pediatric disease, with extraordinary impacts on public health and the world’s economies, identifying and mitigating risks associated with lead exposures should be a priority of international governmental and nongovernmental agencies and programs.

## Supplementary information

Supplementary Information

## Data Availability

Data are available from the corresponding author upon reasonable request.
