# Supplementary material for: Evaluating metal cookware as a source of lead exposure
Source: J Expo Sci Environ Epidemiol. 2024 May 21;35(3):342–50. doi: 10.1038/s41370-024-00686-7 (PMC12069085; doi:10.1038/s41370-024-00686-7)

# Supplementary Information:

**Evaluating metal cookware as source of lead exposure**

## XRF analysis of cookpots

Samples were analyzed in Bruker’s “Restricted Materials” application, using the “Automatic Calibration Selection” method, for 60 seconds (30 seconds for phase 1, and 30 seconds for phase 2).

To ensure that the instrument was appropriately configured to measure the lead content of stainless steel objects, we performed calibration tests at the onset of this study using five standards that contained between 25 ppm and 380 ppm lead in a stainless steel matrix (Brammer Standard Company, Inc., Houston, TX). The five standards were each scanned fifteen times – five times on three different days. The measured concentrations were recorded in Microsoft Excel^TM^ and correlation coefficients were calculated using R (The R Foundation for Statistical Computing, Vienna, Austria). Descriptive statistics for the XRF analyzer calibration test are presented in Table S-1 and a graph showing the XRF analyzer response is presented in Figure S-1. The statistical analysis yielded correlation coefficients of 0.9700 and 0.9179 using the Pearson and Spearman correlation methods, respectively. We conclude that the XRF analyzer used in this study is configured appropriately to measure lead levels in stainless steel over the range of concentrations in the standards.

To verify consistency in instrument response when screening cookpots, a calibration test was conducted before and after screening each cookpot, with five replicate XRF runs using Bruker’s SAC 305 calibration standard.

Element-specific limits of detection (LODs) in XRF analysis are dependent on several factors, including mode of instrument application, counting time, and sample density, composition, and thickness. Measurements are considered to be below the LOD if the value is lower than or equal to the error term associated with the sample. Although the Bruker XRF analyzer does not display a value at or below the element’s LOD, a value is recorded in a sample-specific data file. Therefore, we reviewed the data files for scans below the LOD and used the recorded value in the file to describe the lead concentration in these samples.

*Leachate analysis*

Cookpots were filled to within three inches of the rim with the leaching solution, the lid put in place, and heated. If the cookpot was not equipped with a lid, a silicone covering was placed on the opening. For safety reasons, we did not allow the pressure cookers to pressurize (i.e., we did not secure the lids to create a seal – steam was allowed to escape, unimpeded). After the liquid had boiled for 15 minutes, the cookpot was removed from the heat and a 100 mL aliquot of liquid was transferred to a 250 mL HDPE sample container (Thermo Scientific 316-0250) using a 25-ml capacity serological glass disposable pipet (Fisherbrand^TM^ #13-678-36D). The cookpot was allowed to sit for an additional 24 hours at 20-24°C (68-75°F), when another 100 mL aliquot was removed.

For preservation, 2 mL of concentrated nitric acid (Fisher TraceMetal^TM^ Grade) was added to each 100 mL sample (pH < 2), then sealed with Parafilm® and stored at 4℃ until analysis. Metals analysis was conducted on an Agilent 7900 ICP-MS (Santa Clara, CA) with collision cell technology (He mode). A minimum of a 5-point matrix matched calibration was run for each batch with continuing calibration verification (CCVs) run at least once per 10 samples. Laboratory control samples (LCS) per batch consisted of one low-spike at 1 ppb, one mid-spike at 5 ppb, and two high-spikes at 20 ppb with mean recoveries within 5% for all batches, and an overall project mean of 102 ± 2% for Pb (*N=39*). Sets of duplicate matrix spikes (*N=17*) at 20 ppb were also conducted on samples throughout the project to confirm accuracy and precision. Results indicated good recoveries with a mean at 103% and a relative percent difference below 5% for all batches. All other QC parameters (2^nd^ source check standards, blanks, instrument duplicated) were within the established criteria of the University of Washington, Environmental Health Laboratory QC Program.

**Supplemental figure legends**

Figure S-1. XRF analyzer response to lead-containing stainless steel standards

**Supplemental table legends**

Table S-1. Descriptive statistics for the XRF analyzer calibration test

Table S-1. Descriptive statistics for the XRF analyzer calibration test

| **Stainless Steel Standard** | | | **XRF measurement** | | |
| --- | --- | --- | --- | --- | --- |
| **Name** | **Concentration (ppm)** | **Mean (ppm)** | | **Median (ppm)** | **Standard Deviation** |
| C26 | 25 | 40.7 | | 54 | 27.2 |
| SS 467/1 | 40 | 82.7 | | 78 | 22.0 |
| C1153a | 60 | 98.0 | | 96 | 35.3 |
| C1154a | 170 | 241 | | 233 | 22.6 |
| C25 | 380 | 411 | | 411 | 37.2 |

Figure S-1. XRF analyzer response to lead-containing stainless steel standards


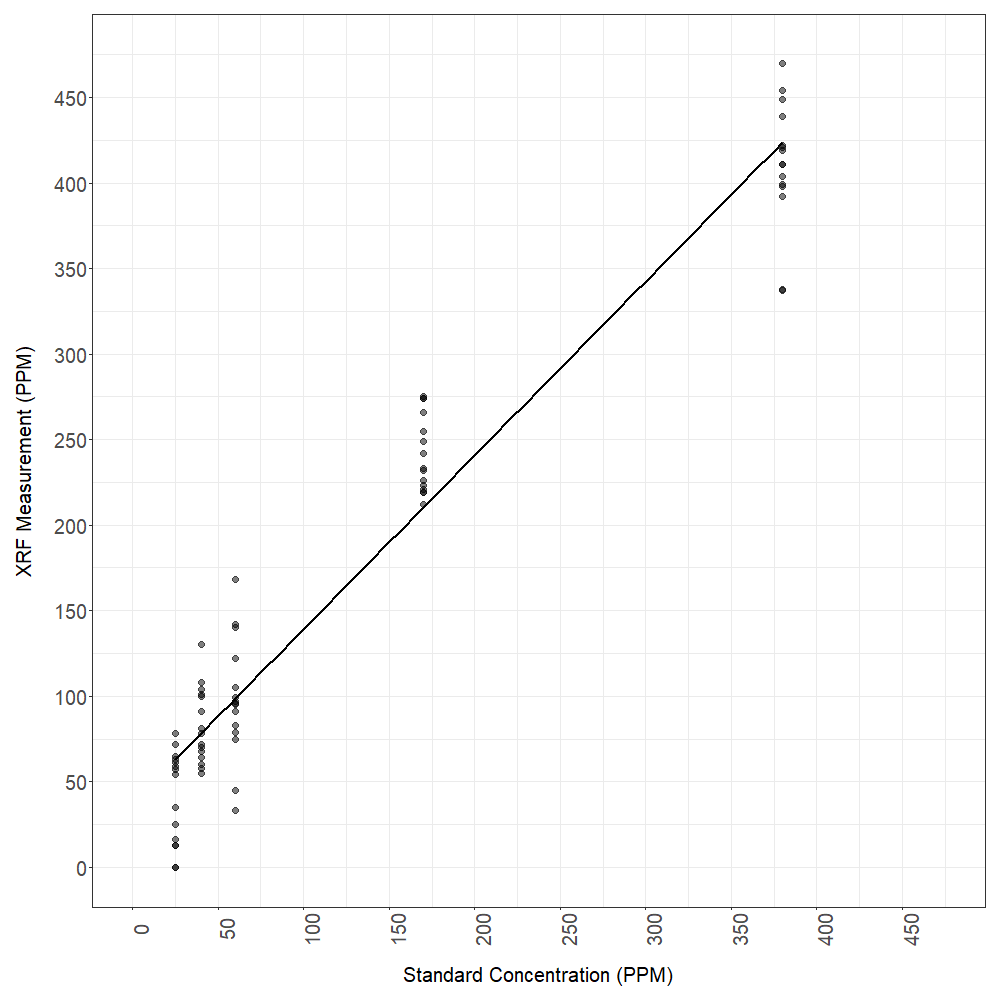

Supplement: Supplementary file 1 — Supplementary Information [file 41370_2024_686_MOESM1_ESM.docx]
